# A prospective, multicentre, registry study of RECO in the endovascular treatment of acute ischaemic stroke

**DOI:** 10.1038/s41598-024-52207-z

**Published:** 2024-01-25

**Authors:** Yunlong Ding, Tingting Zhai, Ronghua Chen, Fangshu Chen, Yanbo Cheng, Shiguang Zhu, Yajie Liu, Guodong Xiao, Yunfeng Zhang, Yan Liu, Zhongrong Miao, Jiali Niu

**Affiliations:** 1 Department of Neurology, JingJiang People’s Hospital, The Seventh Affiliated Hospital of Yangzhou University, Taizhou, China; 2 Department of Neurosurgery, The First People’s Hospital of Changzhou, Changzhou, China; 3 Department of Neurology, Ji’nan Zhangqiu District People’s Hospital, Ji’nan, China; 4 Department of Neurology, The Affiliated Hospital of Xuzhou Medical University (East Hospital District), Xuzhou, China; 5 Department of Neurology, The Affiliated Hospital of Xuzhou Medical University, Xuzhou, China; 6 Department of Neurology, Southern Medical University Shenzhen Hospital, Shenzhen, China; 7 Department of Neurology, Second Affiliated Hospital of Soochow University, Suzhou, China; 8 Department of Neurology, Affiliated Hospital of Nantong University, Nantong, China; 9 Department of Interventional Neuroradiology, Beijing Tiantan Hospital, Capital Medical University, Beijing, China; 10 Department of Clinical Pharmacy, Jingjiang People’s Hospital, The Seventh Affiliated Hospital of Yangzhou University, Taizhou, China

**Keywords:** Diseases, Medical research, Neurology

## Abstract

The RECO is a novel endovascular treatment (EVT) device that adjusts the distance between two mesh segments to axially hold the thrombus. We organized this postmarket study to assess the safety and performance of RECO in acute ischaemic stroke (AIS) patients with large vessel occlusion (LVO). This was a single-arm prospective multicentre study that enrolled patients as first-line patients treated with RECO at 9 stroke centres. The primary outcome measures included functional independence at 90 days (mRS 0–2), symptomatic intracranial haemorrhage (sICH), time from puncture to recanalization and time from symptom onset to recanalization. The secondary outcome measures were a modified thrombolysis in cerebral infarction (mTICI) score of 2b or 3 after the first attempt and at the end of the procedure and the all-cause mortality rate within 90 days. From May 22, 2020, to July 30, 2022, a total of 268 consecutive patients were enrolled in the registry. The median puncture-to-recanalization time was 64 (IQR, 45–92), and the symptom onset-to-recanalization time was 328 min (IQR, 228–469). RECO achieved successful reperfusion (mTICI 2b-3) after the first pass in 133 of 268 patients (49.6%). At the end of the operation, 96.6% of the patients reached mTICI 2b-3, and 97.4% of the patients ultimately achieved successful reperfusion. Sixteen (7.2%) patients had sICH. A total of 132 (49.3%) patients achieved functional independence at 90 days, and the all-cause mortality rate within 90 days was 17.5%. In this clinical experience, the RECO device achieved a high rate of complete recanalization with a good safety profile and favourable 90-day clinical outcomes.

**Clinical trial registration:** URL: https://www.clinicaltrials.gov/; Unique identifier: NCT04840719.

## Introduction

Endovascular treatment (EVT) has been suggested to be a successful vascular reconstruction treatment for acute ischaemic stroke (AIS) due to large vessel occlusion (LVO)^1,2^. A recent study revealed that EVT is also beneficial for AIS patients with large infarcts^3^. Among the various EVT techniques, stent retriever thrombectomy has recently been widely used^4,5^. EVT with stent retrievers for intracranial LVO has been associated with faster and greater rates of reperfusion and good recanalization, ranging from 67 to 94%^6,7^.

The Solitaire FR is the first stent retriever that has been proven to be effective in EVT^8^. Research and development of new stent retrievers is currently a hot topic, with the aim of increasing the efficiency of EVT, reducing complications and ultimately improving patient prognosis. The application of various stents, including Tigertriever^9^, ThrombX^10^, Nimbus^11^, and Trevo^12^, in EVT has been reported to yield favourable outcomes. These stents have demonstrated effectiveness in vascular reconstruction, demonstrating their potential benefits in the context of EVT.

**Figure 1:**
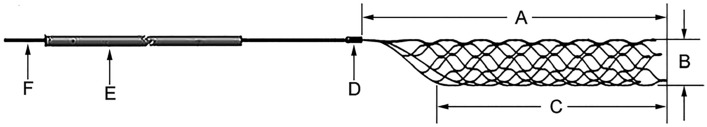
Description of stent composition: total length of stent (**A**), diameter of stent (**B**), length of stent available for use (**C**), radiopaque markers (**D**), protection sheath (**E**), and pushing wire (**F**).

The success of EVT in AIS patients has prompted ongoing efforts to enhance the efficacy of clot retrieval devices. In this context, the RECO stent has emerged as a pioneering clot retriever developed in China. It is utilized primarily in East Asian populations and exhibits distinctive features from those of conventional devices. The stent retriever used was a self-expanding clot-retrieving stent, a pushing wire connected to the stent, and a protection sheath (Fig. 1)^13^. It is a self-expanding clot retriever designed for FR in patients with ischaemic stroke caused by intracranial LVO.

A prospective randomized controlled trial showed that the RECO stent retriever is an effective and safe EVT device for AIS due to LVO^13^. However, RCTs are limited by the restrictive nature of the study design in many aspects, such as the inclusion and exclusion criteria, selection status, and operator experience, and the results may not be generalizable to real-world practice. Therefore, we designed this prospective registration study to evaluate the effectiveness of the RECO device in real-world LVO patients.

## Methods

This was a single-arm prospective multicentre study in which patients were enrolled first-line with RECO at 9 stroke centres between May 22, 2020, and July 30, 2022 (https://www.Clinicaltrials.gov; unique identifier: NCT04840719), a postmarket study assessing the safety and performance of RECO in AIS patients with LVO.

### Inclusion and exclusion criteria

The inclusion criteria for the present study were as follows: (1) ≥ 18 years of age; (2) diagnosis of AIS; (3) imaging-confirmed intracranial LVO: intracranial internal carotid artery (ICA T/L), middle cerebral artery (MCA M1/M2), anterior cerebral artery (ACA A1/A2), basilar artery (BA), vertebral artery (VA V4), and posterior cerebral artery (PCA P1); (3) ASPECT or PC-ASPECT ≥ 6; (4) initiation of any type of EVT, including intra-arterial thrombolysis, mechanical thrombectomy (MT), angioplasty, and stenting; and (5) ability and willingness to sign the informed consent form. Patients who met the following criteria were excluded: had (1) an isolated cervical ICA or VA occlusion and (2) no evidence of LVO on DSA.

### Sampling size

PASS 2021(NCSS, USA) calculated 233 sample sizes using one-sample t-tests for superiority by a margin (power: 0.8, α = 0.1), according to the procedure time from puncture to mTICI reperfusion grade ≥ 2 in REDIRECT study^13^. Considering the dropout rate (10%), 259 sample sizes were recommended. The sample size was determined based on the total number of patients with AIS who received RECO at the selected hospitals during the study period. For this purpose, 268 patients were enrolled in this study.

### Data collection and outcome measures

All variables, including demographic data, medical history, prestroke modified Rankin scale (mRS) score, baseline National Institutes of Health Stroke Scale (NIHSS) score, vital signs, laboratory and neurovascular imaging results, workflow intervals, and clinical outcomes, were prospectively collected.

The primary outcome measures included the 90-day mRS score, functional independence at 90 days (mRS 0–2), symptomatic intracranial haemorrhage (sICH) within 12–36 h after the procedure according to the Heidelberg Bleeding Classification^14^, and the time from symptom onset to recanalization.

The secondary outcome measures were the recanalization rate [defined as a modified Thrombolysis in Cerebral Infarction (mTICI) score of 2b or 3^15^] after the first attempt, the recanalization rate at the end of the procedure, and the all-cause mortality rate within 90 days.

### Statistical analysis

SPSS software (version 26.0; SPSS, Inc., Chicago, IL) was used for the statistical analyses. Continuous/ordinal variables are described as medians (interquartile ranges (IQRs)), and categorical variables are described as numbers (percentages). Univariate analysis was used to determine predictors of clinical outcome. A P value of < 0.05 was considered to indicate statistical significance.

### Ethical approval and consent to participate

Ethics approval was granted by the ethics committees of Beijing Tiantan Hospital (KY 2019-080-03) and all participating centres. The patient management principles at all centres adhered to the standards outlined in the ‘Chinese guidelines for endovascular treatment of acute ischaemic stroke 2018’^16^ and the ‘Chinese guidelines for diagnosis and management of acute ischaemic stroke 2018’^17^. The subjects or their representatives provided written informed consent.

## Results

From May 22, 2020, to July 30, 2022, a total of 268 consecutive patients were enrolled in the registry at 9 stroke centres.

### Demographic and baseline features

The demographic and baseline characteristics are summarized in Table 1. The median age was 69 (IQR, 59–76) years, 166 (61.9%) were male, 77 (28.7%) were transferred from primary stroke centres, the median prestroke mRS was 0 (IQR, 0–0), the baseline NIHSS was 18 (IQR, 13–23), 109 (58.0%) patients had cardiogenic cerebral embolism stroke, and 72 (38.3%) patients had large atherosclerotic stroke. Fifty-nine patients (22.0%) received recombinant tissue plasminogen activator (rtPA) before EVT. A total of 55.3% of the patients received general anaesthesia, and 53.0% of the patients received suction catheters.

**Table 1:** The demographic and baseline characteristics.

Items	N	Median, IQR/*n*, %
Male	268	166 (61.9)
Age, y	268	69 (59–76)
Smoking	245	
Never		196 (80.0)
Current		44 (18.0)
Recent		5 (2.0)
Drinking	261	
Never		217 (83.1)
Occasionally		22 (8.4)
Current		19 (7.3)
Recent		3 (1.1)
Cerebral haemorrhage	263	3 (1.1)
Hypertension	263	174 (66.2)
Diabetes	262	38 (14.5)
Hyperlipidaemia	263	2 (0.8)
Previous stroke	264	57 (21.6)
Coronary heart disease	262	34 (13)
Atrial fibrillation	264	96 (36.4)
TIA	264	7 (2.7)
Heart valve disease	262	12 (4.6)
Interhospital transfer	268	77 (28.7)
PremRS	268	0 (0.0)
0		249 (92.9)
1		11 (4.1)
2		7 (2.6)
4		1 (0.4)
Initial NIHSS	268	18 (13–23)
TOAST classification	188	
Large atherosclerotic stroke		72 (38.3)
Cardiogenic cerebral embolism		109 (58.0)
Other stroke with definite aetiology		2 (1.1)
Stroke of unknown aetiology		5 (2.7)
rtPA	268	59 (22.0)
Anaesthesia	266	
Local anaesthesia		119 (44.7)
General anaesthesia		147 (55.3)
Catheter suction	268	142 (53.0)

TIA, transient ischaemic attack; mRS, modified Rankin scale; NIHSS, National Institutes of Health Stroke Scale; rtPA, recombinant tissue plasminogen activator.

### Workflow intervals

The time metrics are shown in Table 2. The median symptom onset-to-door time was 126 min (IQR, 68–277), the door-to-puncture time was 95 (IQR, 74–130), the symptom onset-to-puncture time was 250 min (IQR, 164–380), the onset-to-recanalization time was 328 min (IQR, 228–469), and the puncture-to-recanalization time was 64 min (IQR, 45–92).

**Table 2:** Workflow intervals.

Items	N	Median, IQR
Onset-to-door time, min	264	126 (68–277)
Door-to-puncture time, min	264	95(74–130)
Onset-to-puncture time, min	264	250 (164–380)
Onset-to-recanalization time, min	254	328 (228–469)
Puncture-to-recanalization time, min	256	64 (45–92)

### Clinical outcomes

The clinical outcomes are shown in Table 3. RECO achieved successful reperfusion (mTICI 2b-3) after the first pass in 133 of 268 patients (49.6%). At the end of the operation, 96.6% of the patients reached mTICI 2b-3, and 97.4% of the patients achieved successful reperfusion. Sixteen (7.2%) patients had sICH. A total of 132 (49.3%) patients achieved functional independence at 90 days, and the all-cause mortality rate within 90 days was 17.5%.

**Table 3:** Clinical outcomes.

Items	N	n, %
sICH	223	16 (7.2)
No. of passes	268	
1		138 (51.5)
2		73 (27.2)
3		37 (13.8)
4		9 (3.4)
5		7 (2.6)
6		3 (1.1)
7		1 (0.4)
First pass mTICI 2b-3	268	133 (49.6)
0		85 (31.7)
1		17 (6.3)
2a		33 (12.3)
2b		45 (16.8)
3		88 (32.8)
Final reperfusion	266	259 (97.4)
Final mTICI	265	256 (96.6)
0		4 (1.5)
2a		5 (1.9)
2b		57 (21.5)
3		199 (75.1)
90d-mRS	268	
0		78 (29.1)
1		31 (11.6)
2		23 (8.6)
3		47 (17.5)
4		28 (10.4)
5		14 (5.2)
6		47 (17.5)

*sICH* symptomatic intracranial haemorrhage, *mTICI* modified thrombolysis in cerebral infarction, *mRS* modified Rankin scale.

### Predictors of clinical outcomes

**Figure 2:**
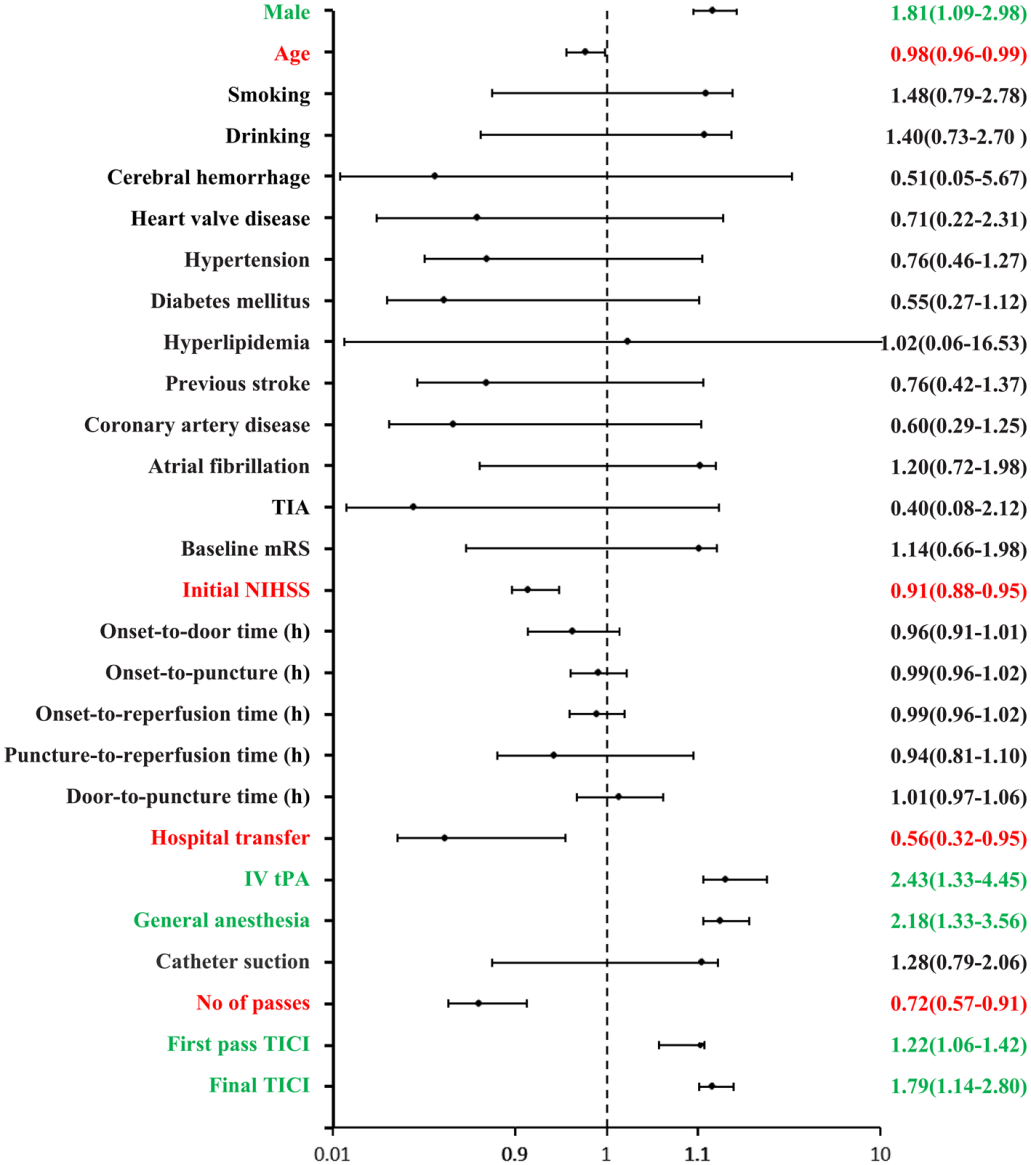
Forest plot of univariate predictors of clinical outcome dichotomized as mRS 0–2. ORs (95% CIs) are depicted to the right of the figure. Red and green bars indicate variables that are significant.

Univariate analysis revealed that male sex, rtPA, general anaesthesia, a higher mTICI after the first pass, a higher mTICI at the end of the operation, younger age, direct admission, a lower initial NIHSS score, a shorter revascularization time, and a smaller number of passes were protective factors for a good clinical outcome (Fig. 2).

## Discussion

To date, this is the largest investigator-initiated, real-life, postmarketing, clinical registry evaluating a total of 268 patients treated with the RECO device (Minitech Medical) as the first clot retriever in China. Our study revealed angiographic and clinical outcomes similar to those reported in the RECO RCT conducted by Cao Jie et al.^13^, in which the RECO device had good efficacy and safety profile when used in AIS due to LVO, achieving high reperfusion rates and favourable clinical outcomes.

Functional independence (mRS score ≤ 2) at 90 days is considered the gold standard for assessing the clinical outcome of AIS. Our study showed that 49.3% of the patients had an mRS score ≤ 2 at 90 days. Piotr Piasecki et al. reported that 35% of the patients in that study had a good prognosis according to the Tigertriever device^9^; Osama O Zaidat et al. reported a 47.9% good prognosis according to the TREVO Stent-Retriever^12^; and the good prognosis rate ranged from 36 to 46%^18–20^ . This finding confirms the effectiveness of the RECO device in the real world; however, these data are lower than the 62.7% good prognosis rate of the RECO stent group in the RECO RCT study^13^. We suspect that this difference may be related to the stricter admission criteria used in the RCT study. We identified several factors that may contribute to the outcome disparities in these two studies: (1) in the RCT, the patients included had AIS within 8 h after symptom onset, whereas our study did not impose restrictions on the onset time. (2) In the RCT, patients with a baseline NIHSS score ≥ 8 or ≤ 24 were included; in our study, some patients had an NIHSS score exceeding 24, indicating a poorer prognosis. (3) An RCT reported an IVT rate of 81% in the RECO stent group, which was significantly greater than that in our study (22%) and could be a crucial influencing factor on outcomes. (4) The age of the patients in the RCT study's RECO stent group was 61 years, which was notably lower than that in our study's 69 years, suggesting that a younger age might contribute to better outcomes. We plan to conduct a thorough analysis of these factors in future investigations to gain a comprehensive understanding of their impact on the outcomes of patients undergoing RECO stent EVT.

We observed that 49.6% of the patients reached mTICI 2b-3 after the first pass of the RECO device, which was significantly greater than the 41% reported in other studies^19^. In addition, at the end of the operation, 96.6% of the patients ultimately reached an mTICI of 2b-3, and the proportions of patients with other reported embolization devices and a final mTICI rating of 2b-3 were 71–89%^12,18,20–23^. As the first clot retriever in China, the RECO stent is primarily used for cerebrovascular diseases in East Asian people and has several notable advantages. First, RECO utilizes mechanical fastening to reinforce the connection between the stent and the push wire, enhancing the pushability and safety of device withdrawal. Second, the design of the struts of the RECO stent has undergone optimization, which has resulted in a slightly greater radial force than that of other devices. This contributes to a higher success rate of recanalization. Additionally, the RECO stent offers more options for diameter and length, catering to intracranial vessels of various sizes. This versatility may account for its advantage in terms of vascular reflux rates. With a higher reperfusion rate, the median procedure time from puncture to an mTICI reperfusion grade ≥ 2b was 64 min, which was significantly lower than that of other devices. This finding suggested that the RECO device demonstrates efficient handling and embolization capabilities.

The use of suction catheters may increase the efficiency of stent embolization and reduce the risk of distal embolization^22^. Fifty-three percent of the patients in our study received suction catheters. However, the use of suction catheters is not a protective factor for good prognosis, which suggests that the prognosis obtained by using the RECO device alone is not inferior to that of suction catheters combined with stents. Previous research has confirmed that failure to achieve successful recanalization is associated with different variables, such as the composition and length of the clot^24^; vascular tortuosity^25^; and procedural factors, such as clot fragmentation, distal embolization^26^ or early reocclusion^27^.

After analysing the risk factors for a good prognosis, we observed that venous thrombolysis can improve patient prognosis. However, in this study, only 59 patients (22.0%) received rtPA before EVT. We believe the possible reasons are as follows: (1) upon analysing the data, we observed that 73 patients were admitted more than 4.5 h after symptom onset, rendering them ineligible for intravenous thrombolysis (IVT). (2) Patients admitted within the 4.5-h window might have undergone direct EVT due to contraindications for IVT. (3) An inevitable factor is bias among physicians towards IVT. The 2020 DIRECT-MT study revealed that direct EVT was noninferior to IVT bridging EVT, potentially influencing practitioner choices. On the basis of our centre's experience, some patients deemed at higher risk of bleeding might opt for direct EVT. Indeed, biases against IVT should be acknowledged and addressed because they play a significant role in clinical decision-making. Recently, six phase III randomized controlled trials (RCTs) have attempted to demonstrate the noninferiority of direct mechanical thrombectomy (dMT) compared to bridging IVT followed by thrombectomy [bridging alteplase therapy (BT)] in AIS patients, and the study yielded inconsistent results^16,24–26,28,29^. Our study suggested that IVT is currently necessary for suitable patients before using the RECO device for MT.

Currently, it is debated whether the type of anaesthetic technique used during MT has a relevant impact on neurological outcomes. A meta-analysis showed that general anaesthesia may improve adverse events (haemodynamic instability) compared to nongeneral anaesthesia, with low-certainty evidence. However, there was no evidence of a difference in neurological impairment, stroke-related mortality, intracranial haemorrhage or haemodynamic instability adverse events between groups with low-certainty evidence^30^. Our research has shown that general anaesthesia can improve the prognosis of patients. This may provide a reference for anaesthesia options during MT, but additional randomized controlled trials with a low risk of bias are still needed to reduce our uncertainty and to aid decision-making in the choice of anaesthesia.

We observed that age, hospital transfer, and the number of embolizations were risk factors for poor prognosis. The older the patient is, the greater the risk of complications, and an increase in the number of embolizations prompts greater surgical difficulty and longer puncture-to-recanalization time, which may be the cause of poor prognosis. Patients who are referred have a higher rate of adverse prognosis. Our previous studies revealed the impact of referral on the green channel process in ischaemic stroke patients. Patients who are referred will have a longer onset-to-recanalization time due to prehospital delays, which leads to a poor prognosis. Therefore, it is necessary to construct an efficient green channel regional network to improve the prehospitalization process in stroke patients.

There are several limitations in our study. First, this prospective multicentre study included AIS patients from 9 centres. The stroke green channel processes in these 9 centres are not the same, which may affect the analysis and evaluation of patient prognosis. Second, we conducted a risk factor analysis of patient prognosis, but the impact of these risk factors on patient prognosis requires prospective randomized controlled studies designed for each factor to reduce selection bias.

## Conclusion

This multicentre, prospective study suggested that the RECO device has a good efficacy and safety profile when used in AIS due to LVO, achieving high reperfusion rates and favourable clinical outcomes.

## Data Availability

The datasets used and analysed during the current study are available from the corresponding author upon reasonable request. The requests to access the datasets should be directed to Zhongrong Miao, Department of Interventional Neuroradiology, Beijing Tiantan Hospital, Capital Medical University; email: zhongrongm@163.com.
